# Agroecological Service Crops Drive Plant Mycorrhization in Organic Horticultural Systems

**DOI:** 10.3390/microorganisms9020410

**Published:** 2021-02-16

**Authors:** Alessandra Trinchera, Elena Testani, Giancarlo Roccuzzo, Gabriele Campanelli, Corrado Ciaccia

**Affiliations:** 1 Consiglio per la Ricerca in Agricoltura e l’analisi dell’economia Agraria. Centro di ricerca Agricoltura e Ambiente (CREA-AA), Via della Navicella, 2, 00184 Rome, Italy; elena.testani@crea.gov.it (E.T.); corrado.ciaccia@crea.gov.it (C.C.); 2 Consiglio per la Ricerca in Agricoltura e l’analisi dell’economia Agraria. Centro di ricerca Olivicoltura, Frutticoltura e Agrumicoltura (CREA-OFA), Corso Savoia 190, 95124 Acireale, Italy; giancarlo.roccuzzo@crea.gov.it; 3 Consiglio per la Ricerca in Agricoltura e l’analisi dell’economia Agraria. Centro di ricerca Orticoltura e Florovivaismo (CREA-OF), Via Salaria, 1, 63074 Monsampolo del Tronto, Italy; gabriele.campanelli@crea.gov.it

**Keywords:** mycorrhizal fungi, mycelial network, weed traits, cereal cover crop, intercropping, organic melon, organic orange, electron scanning microscopy

## Abstract

Mycorrhizal symbiosis represents a valuable tool for increasing plant nutrient uptake, affecting system biodiversity, ecosystem services and productivity. Introduction of agroecological service crops (ASCs) in cropping systems may determine changes in weed community, that can affect the development of the mycorrhizal mycelial network in the rhizosphere, favoring or depressing the cash crop mycorrhization. Two no-till Mediterranean organic horticultural systems were considered: one located in central Italy, where organic melon was transplanted on four winter-cereals mulches (rye, spelt, barley, wheat), one located in southern Italy (Sicily), where barley (as catch crop) was intercropped in an organic young orange orchard, with the no tilled, unweeded systems taken as controls. Weed “Supporting Arbuscular Mycorrhiza” (SAM) trait, weed density and biodiversity indexes, mycorrhization of coexistent plants in the field, the external mycelial network on roots were analyzed by scanning electron microscopy, crop P uptake, yield and quality were evaluated. We verified that cereals, used as green mulches or intercropped, may drive the weed selection in favor of the SAM species, and promote the mycelial network, thus significantly increasing the mycorrhization, the P uptake, the yield and quality traits of the cash crop. This is a relevant economic factor when introducing sustainable cropping practices and assessing the overall functionality of the agroecosystem.

## 1. Introduction

Nowadays, considering the growing world demand for long-term sustainability of cropping systems and healthy food, organic agriculture represents the most promising management; when relying on agroecological principles, such as crop diversification, the reduction in external inputs, the introduction of agroecological service crops (e.g., cover crops, living mulch), and the maintenance of natural biodiversity [1,2,3,4]. Considering the growing demand for organic products, especially in EU countries, the organic farmers ask for new, effective tools compatible with organic management, which may guarantee adequate crop yields and quality [5].

Although a number of plant families are not mycorrhizal, like *Brassicaceae,* in horticultural crop production the mycorrhizal symbioses represent a very efficient tool for facilitating the plant nutrient uptake and crop productivity [6,7]. Indeed, crop mycorrhization improves P and N uptake and use efficiency, allowing reduction in fertilizer inputs [8,9,10]. Mycorrhization is also able to increase the horticultural crop quality through improving their ability to front abiotic and biotic stresses, as observed in mycorrhizal-inoculated melon and tomato [11,12].

An alternative approach to mycorrhizal bioinoculant application is based on promoting the spontaneous plant–mycorrhizal symbiosis exploiting specific agroecological practices able to boost the root–fungi rhizosphere interaction, particularly in horticultural crop production.

The mycorrhizal association develops through a complex interaction among mycorrhizal fungi and a plant community, the external mycorrhizal mycelial network having a fundamental role, modulating plant–plant interactions, playing a socio-ecological role in all-natural environments [13]. If this ability to interconnect different individuals is widely recognized in arboreal systems, it is much less so in herbaceous systems, where endomycorrhizas do not always develop external mycelium capable of spreading, connecting plants of different species. However, in diversified agricultural systems, the role played by the mycorrhizal network among coexisting plants, such as the yield crops and the spontaneous flora (i.e., the weeds), has been observed [14], although it is only understood partly so far.

In the field, different herbaceous species interact above and belowground, sharing their roots, and selecting each other’s by interference [1], so to be deeply influenced by reciprocal ability to be colonized, or not, by arbuscular mycorrhizal fungi (AMF). Intensive and frequent ploughing, as the main conventional tillage practice, changes profoundly the composition of soil biota communities, and lowers abundance and diversity of wild plants and beneficial soil organisms [15,16]. Fungi are particularly affected by soil tillage more than bacteria, since their large hyphal networks are disrupted by tillage, thus depressing plant–fungi symbioses [17]. Thus, in no-till organic crop rotations, the use of agroecological service crops (ASCs, i.e., all the plant species in a system which are grown not for yield purposes but to provide ecosystem services to the agroecosystem) intercropped, or as cover crops to form a green mulch after termination, is a profitable agronomic strategy to contain weed emergences, avoiding disturbance of microorganisms living in the upper soil layer, including mycorrhizal fungi [18,19,20,21,22].

The ASC introduction inevitably determines a selection of mycorrhizal or non-mycorrhizal weed species that can affect the development of the mycelial network in the rhizosphere, thus favoring or depressing the mycorrhization of the cash crop. In an artichoke organic production, a selected intercropped living mulch, compared to the same no mulched system, was able not only to contain weeds, but also to promote artichoke mycorrhization [10,23]. In a no-till, Mediterranean organic rotation, the introduction of rye and spelt ASCs as winter-cereal cover crops inhibited or boosted the spontaneous mycorrhization of coexistent plants, respectively [14]. In fact, due to their allelopathic properties, the rye exudates were able to inhibit the radicle development of the curly dock, thus hindering the mycorrhizal colonization at the beginning of the seedling, preventing the AMF intra-hyphal invagination in cortex cells of the weed rootlet [24].

Moving onto fruit trees, most of them exhibit a mycotrophic habit, thus a high benefit can be obtained by enhancing the AMF colonization of their roots [25]. The benefits of green mulching on soil microbiota were already observed in pomelo in improving the soil microbial growth and AMF root colonization in an organic orchard [26]. In Tarocco orange trees, the positive effect of composted residues on secondary root proliferation and mycorrhization was observed [27]. More recently, the mycorrhizal bioinoculation increased arbuscular mycorrhization in citrus trees by enlarging the contacted areas of roots to soil, thereby promoting nutrient uptake and supporting citrus plants in overcoming abiotic and biotic stresses [28]. However, to date no information is available on the effect of ASC introduction as living mulch in organic citrus orchards on root mycorrhization, mediated by weed selection.

Based on above-described ASC ability to select weeds, we hypothesize that: (i) introduction of ASCs may increase or reduce the proportion of mycorrhizal weeds; (ii) the composition and functional traits of the selected weed community may influence the mycorrhization of the cash crop, an effect not yet sufficiently investigated [29,30]. Consequently, we identified the weed mycorrhizal trait as a key factor, although it is rarely inserted in plant traits databases and almost totally neglected in trait-based plant community studies.

This study focused on two Italian no-till, organic horticultural systems, where different used winter cereals ASCs were introduced to form green mulches or intercropped. The weed selection and traits driven by the ASC, the effect of mycorrhizal weeds on mycorrhization, nutrient uptake, yield and/or quality of the cash crop were evaluated.

## 2. Materials and Methods

### 2.1. Experiments Setup and Treatments

Aiming at verifying if agroecological service crops’ introduction may drive cash crop mycorrhization via weed selection; in 2014 two case studies were considered.

#### 2.1.1. Case-Study on Organic Melon

The first experiment, located in Central Italy, consisted of in an organic rotation where the melon (*Cucumis melo* L., cv HF1 Anish) was transplanted onto different winter-cereals mulches. The study was carried out in 2014 at the “MOnsampolo VEgetables organic Long-Term Experiment” (MOVE LTE) at CREA—Consiglio per la Ricerca in Agricoltura e l’analisi dell’economia agraria, located in Monsampolo del Tronto (AP) (latitude 42°53′ N, 13°48′ E), in the coastal area of the Marche Region, Central Italy. The MOVE LTE was established in 2001 and it is based on a 4-year crop rotation with six main crops and three ASCs (as break crops). Additional information about the MOVE LTE is available in Campanelli and coll. [21]. The site is characterized by a “thermo-Mediterranean” climate [31], with an average total annual precipitation of 564 mm and average temperatures ranging about 9 °C in the October–March and 20 °C in April–September periods, respectively. According to Soil Taxonomy of the U.S. Department of Agriculture [32], the soil at the field trial site was Typic Calcixerepts fine–loamy, mixed thermic.

The experimental design was a randomized block design (RBD) with 3 replications (blocks) and one factor, assigned to the ASC treatment (Yes ASC). Five ASC treatments were compared: (i) wheat (*Triticum aestivum* L.); (ii) barley (*Hordeum vulgare* L.); (iii) rye *(Secale cereale* L.); (iv) spelt (*Triticum dicoccum* L.); (v) tilled fallow as control (No ASC). The ASCs were sown on 31 October in 2013 at a rate of 250 kg ha^−1^ in each plot (18 m^2^). The ASCs were terminated at full flowering stage on 29th April, by using an In-Line tillage Roller Crimper, which allowed the flattening of the ASCs and simultaneous preparation of the transplanting furrows for the following crop in the rotation (i.e., melon), guaranteeing no till in the inter-row spaces [25]. In the fallow plots (No ASC), soil was ploughed to a depth of 20 cm at the end of October 2013. Before melon transplanting, control plots were tilled with a rotary tiller (DL 2500; Maschio SPA, Padua, Italy) at a 15-cm depth, contemporary to ASC termination. Melon seedlings were 25 days old and were hand-transplanted at an inter-row × row distance spacing of 1.0 × 1.0 m (1.0 pp m^−2^) on 30th and 18th May. The melon harvest started on the 1st and was completed on the 13th of August 2014, with a cropping cycle of 75 days. Melon crop was irrigated with 1200 m^3^ha^−1^. At melon transplanting, all the trial plots were fertilized using off-farm fertilizers allowed in organic farming according to the European regulation in force, corresponding to 50 kg ha^−1^ of N. Additional amounts of 10 and 8 kg ha^−1^ of N and K_2_O, respectively, were distributed by fertigation along the cropping cycle. No weeding was performed during the ASC and melon cycles. Three melon reference plants per each treatment were considered, for a total of 15 plants (5 system managements × 3 replicates).

#### 2.1.2. Case-Study on Young Organic Citrus Trees

The second experiment, located in Southern Italy, consisted of a organic young orange orchard with barley (as catch crop) intercropping.

The study was carried out in 2014 at the “Long term trial on organic Citrus” (PALAP9, latitude 37°17′ N, longitude14°50′ E) within the experimental farm of the Research Center for Olive, Fruit and Citrus Crops of CREA, in Sicily (Italy). The soil is classified as Eutric Cambisol [33], and the climate of the Region is typical Mediterranean [34], with hot and dry summers. Annual mean reference rainfall is about 500 mm, and the maximum temperature in summer during daytime often reaches 38–40 °C; in 2014 rainfall was 392 mm. The experiment was realized in a young orange orchard: orange trees (*Citrus sinensis* (L.) Osb.) cv. “*Tarocco Rosso*” grafted on Carrizo citrange rootstock (*C. sinensis* (L.) Osb. × *Poncirus trifoliata* (L.) Raf.), planted in June 2012 in single rows spaced 4 by 6 m apart (416 trees × ha^−1^). Trees were drip irrigated using two surface lateral pipes per tree row, with six 4 L h^−1^ emitters (spaced 0.6 m) per tree, 0.35 m from the trunk. The experimental layout was a criss-cross design with three randomized blocks. The vertical strip was assigned to the system management, with the introduction of barley (*Hordeum vulgare* L. cv. Tazio) as agroecological service crop species to contain weeds (Yes ASC), compared to the control, without ASC (No ASC). Fertilization was realized at the dose of 50 kg N per tree of on-farm citrus by-products compost [35]. The barley was sown on 24th November 2013 and on 16th April 2014 terminated by the In Line Roller Crimper (ILRC) technique to form a green mulch to contain weeds. Six reference plants per each treatment were considered, for a total of 12 orange trees (2 system managements × 6 replicates).

### 2.2. Determinations

In both the case-studies, weed community composition was evaluated:

- in the organic melon experiment, by sampling at melon ripening at the 47th day (June 2014) and at melon harvesting on the 56th (August 2014) day after transplanting (DAT), respectively. Total and by-species weed density (pp m^−2^) and cover (%, m^2^ /m^2^) were recorded by placing three randomly selected 0.25 × 0.25 m^2^ (June) and 1.0 × 1.0 m^2^ quadrats (August) within each plot.
- in the orange young trees experiment by sampling at barley boot (on 3rd February) and termination (on 16th April 2014), respectively. The total and by-species weed density were recorded by placing twelve randomly selected 1.0 × 1.0 m^2^ quadrats within each plot.

In both the experiments, the weed biodiversity was evaluated by calculating diversity indices, namely species Richness, as number of species per treatment/plot, (R) and the Shannon–Weaver index (H), calculated as:

(1) $$H\;=\;-\sum^{}(Pi\;\times \;lnPi)$$

where “*Pi*” is the proportion of a given species relative to the total number of species found in the *i*-th sample; “*ln Pi*” is the natural logarithm of *Pi*.

Lastly, the frequency of the weed species with “Supporting Arbuscular Mycorrhiza” trait (SAM%) [36] was determined in both the experiments.

In both the case-studies, the in-field crop root mycorrhization was evaluated as follows:

- in the organic melon system, for each treatment and each field replicate, root mycorrhizal colonization intensity (M%) was determined on melon and on three selected and most representative SAM weed species, *Rumex crispus* L. (RUMCR), *Polygonum aviculare* L. (POLAV) and *Anagallis arvensis* L. (ANGAR). The root apparatus of melon and weeds was sampled from the field by using stainless steel cylinders of 6 cm diameter and 20 cm length [10]. For each species, three root subsamples per plot were collected, then pooled to obtain n.1 root sample × n. 3 treatments × n. 3 blocks.
- in young organic orange trees, for each treatment and each field replicate, root mycorrhizal colonization intensity (M%) was determined on orange tree roots, by using stainless steel cylinders of 9-cm diameter and 30-cm length, to obtain n. 2 root samples × n. 2 treatments × n. 3 blocks.

Collected melon and orange tree roots were immediately separated from the soil by washing in distilled water in a sieve of 0.5-mm mesh, and then ordinated into first, second and third-order lateral roots for further analyses. At random, from each pooled “plot” root apparatus of the melon and the three weed species, a total of 10 × 1 cm root pieces per plant (third-order lateral roots, diameter < 2mm) were cut from 5 mm to 15 mm from the root tip by a razor blade. The root fragments were stained by a solution of 0.05% *w/v* methyl blue in lacto-glycerol (1:1:1 lactic acid, glycerol and water) for 1 min and distained by distilled water for 1 min more [37]. Then, the root fragments were placed on grinded slides, mounted in a drop of glycerol, and observed under a light microscope (Nikon E100). The mycorrhizal colonization intensity (M%) of the melon and weed roots was assessed by applying the method of Trouvelot et al. [38], based on the observation of the root fragments occupied by AMF structures. Quantitative data on AMF arbuscular richness recorded on roots of melon, weeds and orange trees were collected to verify if the observed external mycelium on roots was due to AMF colonization (here not reported).

To verify the presence of AMF extra-hyphal (*hyp*) mycelium development on melon and orange tree roots, selected fresh root fragments were observed at increasing magnifications by Scanning Electron Microscopy (Microscope EVO MA10—Zeiss) under variable pressure, equipped with a LaB_6_ electron sources by using the back-scattered electrons detector (SEM-BSE). The applied variable pressure mode (at 20–25 kV EHT/10 Pa chamber pressure) prevented surface damage of such biological and non-conductive samples, giving a high-resolution image without any sample pretreatment [14].

For both the case-studies, the following parameters were selected to evaluate crop quality and growth:

- in the organic melon case-study, at harvest (13th August 2014), melon yield (Mg ha^−1^), fruit average weight (kg) and sugar content (Brix) were determined to evaluate the cash crop production and quality.
- in the organic orange trees case-study, after one month from ASC termination (12th May), the Soil Plant Analysis Development (SPAD) value, the foliar P content (g kg^−1^), the plant height (cm), the canopy diameter (cm) and the canopy volume (m^3^) were determined on all sampled trees.

### 2.3. Statistical Analysis

All tested parameters were statistically analyzed by ANOVA, considering the block (B) as random factor and the treatment (ASC) as fixed factor. Mean comparison was carried out according to a post hoc Tukey’s honestly significant difference (HSD) test using SPSS (IBM Corp., Armonk, NY, USA). On both the melon and orange datasets collected in April and August 2014, respectively, a preliminary correlation analysis was performed between M% and parameters considered in the two case-studies (SAM%, R, H, crop yield and quality, growth parameters):

(2) $$\mathrm{Correl}\;(X,Y)\;=\;\frac{\sum^{}\;\left(x-\;\overset{ˉ}{x}\right)\left(y-\overset{ˉ}{y}\right)}{\sqrt{\sum^{}{\left(x-\overset{ˉ}{x}\right)}^{2}\;\sum^{}{\left(y-\overset{ˉ}{y}\right)}^{2}}}$$

where *x* and *y* are the sample means average (dataset 1 and dataset 2).

Regression models were used to fit the data, considering predicted R-squared values (Excel, ver 1908—Microsoft Office 365 software package 2020).

Then, Principal Components Analysis (PCA) was applied to both datasets. For the case study on organic melon the PCA was run at the August sampling time on a total of 14 variables (9 main weed species cover, the H and R diversity indices, M%, melon yield and Brix), calculated for 15 treatments (No ASC, Barley, Spelt, Rye and Wheat). For the case study on young organic citrus trees, the PCA was run at the April sampling time on a total of 20 variables (12 main weed species cover, the H and R indices, M, foliar SPAD and P, plant height (PH), canopy volume (CV) and trunk circumference (TC)) calculated for 12 treatments (No ASC and Barley). For both the cases, a biplot considering the two most important components was generated with the aim to identify the latent relationship between cases (treatments) and variables. The direction and length of the arrows in the biplot indicate the direction and magnitude in which each variable contributes to the location of each case in the plot. The angle between each arrow and the axes is inversely proportional to the correlation between each variable and the axes constructed in the biplot. The PCA was performed in R 3.5.1 [39] with the “FactoMineR” [40] and the “factoextra” [41] packages.

## 3. Results

### 3.1. Case-Study on Organic Melon

In Table 1, the list of all weed species found in the No ASC/Yes ASC systems in June and August 2014, and related ecological characterization (including Supporting Arbuscular Mycorrhizal trait, SAM), are reported.

In the melon case-study, of a total of 19 weed species found in field, only three were recognized as non-mycorrhizal ones. To evaluate the effect of weed species on melon mycorrhization during its cropping cycle, in Table 2 the frequency of the mycorrhizal weed species (SAM%), the weed density (pp m^−2^), the weed species Richness (R) and the Shannon index (H) determined both in June and in August 2014, the mycorrhizal colonization intensity (M%) of the harvested melon, the melon yield, the average fruit weight and the melon sugar content are reported.

In June, after one month from melon transplanting, % mycorrhizal species and species Richness were not significantly affected by ASC introduction. As expected, weed density was significantly higher (+43%) in No ASC system with respect to the Yes ASC ones, particularly under rye and spelt, while Shannon–Weaver index was significantly higher in Yes ASC systems (1.36) than in No ASC one (0.90). At melon harvest, the ASC effect on weed cover was not significant, while the % mycorrhizal weeds increased strongly in Yes ASC systems (83.6%), compared to the No ASC one (40.8%), although no significant differences were recorded among the different ASCs. Anyway, in August, in all Yes ASC systems the % mycorrhizal species value was higher (from 73.6% to 91.8%) than that of the No ASC one. Species Richness and Shannon–Weaver index were also significantly increased in Yes ASC systems with respect to the No ASC one: in particular, the R parameter resulted more sensitive to ASC species, being significantly higher in wheat system then in the rye and spelt ones. At harvest, the melon M% was significantly increased by the ASCs of about 54%. As in species Richness, the role played by the different ASCs in cash crop mycorrhization was again observed: the melon mycorrhization was 106% higher in the wheat system with respect to the No ASC one, while those recorded in spelt and barley ones were 30% and 10% higher than the control system, respectively. Melon yield was significantly higher in ASC treatments with respect to the No ASC one, although no differenced were recorded among the different ASCs. Fruit weight was not influenced by the ASC introduction, while melon sugar content increased 9.4% in Yes ASC with respect to the No ASC one (*p* < 0.05).

**Figure 1:**
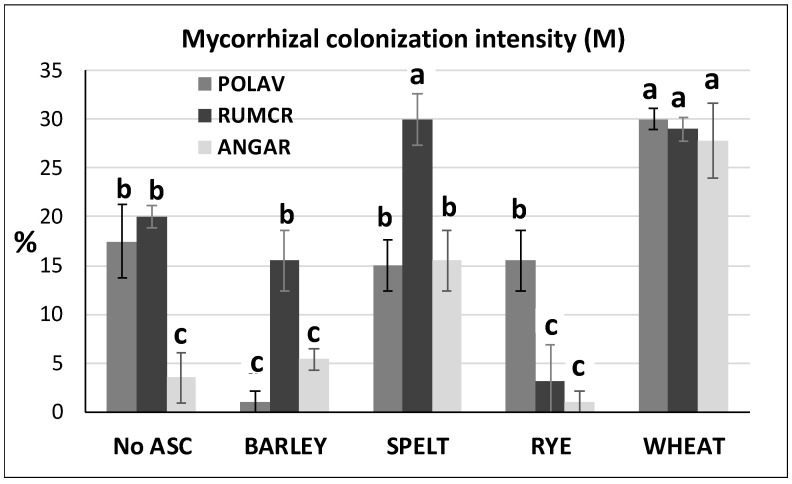
Mycorrhizal colonization intensity (M, in %) of Polygonum aviculare (POLAV), Rumex crispus (RUMCR) and Anagallis arvensis (ANGAR) in No ASC (control) and Yes ASC (barley, spelt, rye and wheat) systems, sampled at melon harvest (August 2014). Different letters represent significant differences (Tukey’s HSD test for means comparison).

In Figure 1, the M% of POLAV, RUMCR and ANGAR sampled at melon harvest in No ASC and Yes ASC systems are reported.

All the considered mycorrhizal weed species showed the highest % mycorrhization in the wheat system, the ANGAR showing a mycorrhization even ten times higher compared to that recorded in rye. In the remaining systems, each mycorrhizal weed showed different mycorrhizal colonization intensity: POLAV was significantly more mycorrhized in No ASC, spelt and rye systems with respect to the barley one, while RUMCR was significantly less mycorrhized in rye, M% being the highest in spelt. ANGAR mycorrhization was significantly lower in No ASC, barley and rye systems compared to the spelt one. However, all the considered mycorrhizal weeds gave the highest mycorrhization in wheat system (*p* < 0.0004), being the ANGAR the M% value even ten times higher compared to that recorded in rye.

**Figure 2:**
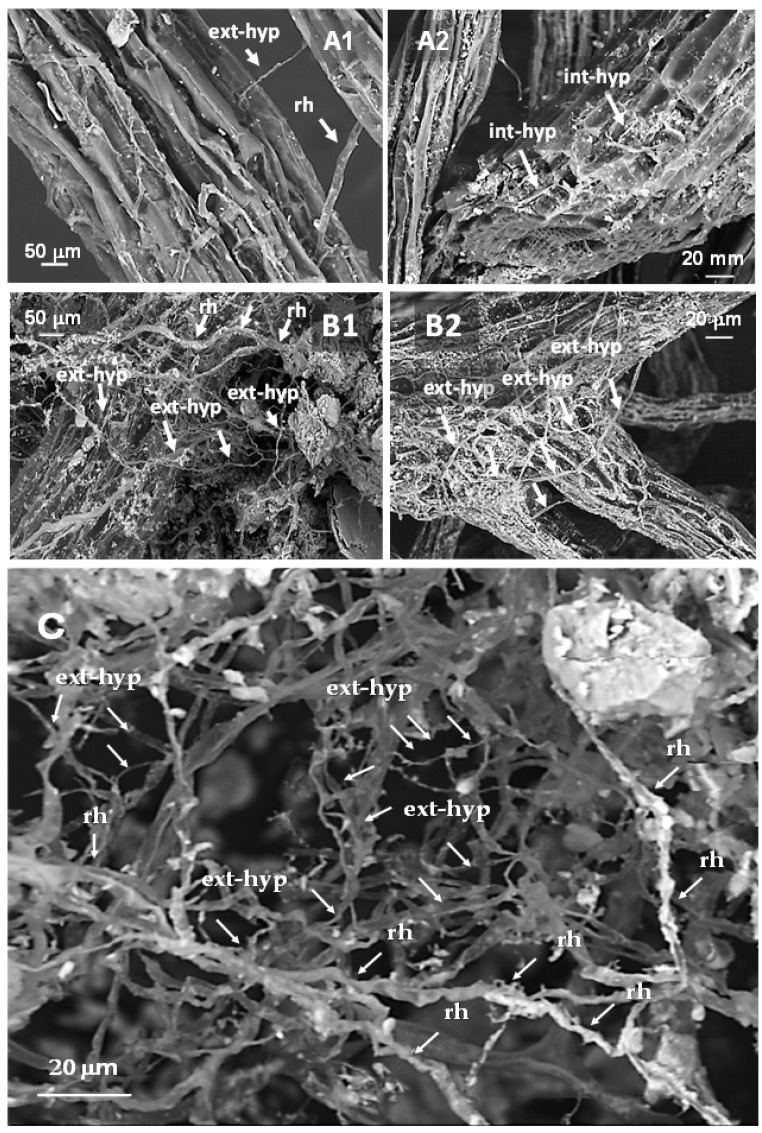
SEM-back-scattered electrons detection (BSE) images of fine lateral roots of *Cucumis melo* in No agroecological service crops (ASC) (**A1,A2**) and Yes ASC-wheat (**B1,B2**) systems, and mycorrhizal network developed on melon roots grown on flattened wheat (**C**). int-hyp: AMF internal hyphae; ext-hyp: AMF extra-radical hyphae; rh: root hairs. **A1**–**B1**: magnification = 200×; **A2**–**B2**: magnification = 700×; C: magnification = 1.5K×.

Electron Scanning Microscopy (SEM-BSE) of melon fine lateral roots collected in No ASC and wheat systems is shown in Figure 2.

An abundant mycelial network surrounding melon roots was observed in the wheat system (Figure 2B1), and it was better highlighted at the highest magnitude (Figure 2B2,C, Video S1). The same mycelial network was poorly developed in the control system, where ASCs were not introduced (Figure 2A1,A2). 

**Figure 3:**
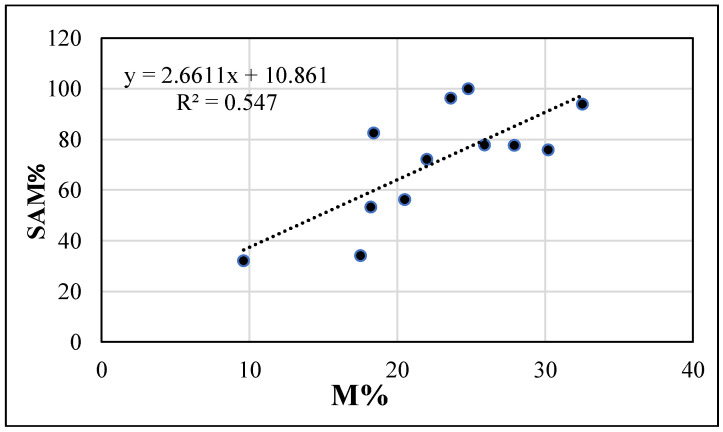
Regression models and *R*^2^ of mycorrhizal colonization intensity (M, in %) vs. frequency of the “Supporting Arbuscular Mycorrhiza” weed species (SAM, in %) in melon systems (No ASC and Yes ASC). Data are refer August 2014 sampling (at melon n harvest, N = 15).

In April, no correlations were found between melon mycorrhization and % mycorrhizal weeds (−0.2684), species Richness (0.2207) or Shannon–Weaver index (−0.3573). On the contrary, at melon harvest, both the % mycorrhizal weeds and the species Richness were highly correlated with the melon mycorrhizal colonization (0.7396 and 0.7945, respectively), corresponding to a positive linear regression between M% and SAM% (*R*^2^ = 0.547, Figure 3). A good correlation was also found between cash crop mycorrhization and Shannon–Weaver index (0.6688). Among the considered mycorrhizal weeds in the field, the POLAV was the most correlated with melon mycorrhization (0.8041), while ANGAR (0.4644) and RUMCRI (0.1593) were poorly correlated.

As far as the melon quality parameters are concerned, crop mycorrhization and sugar content (Brix) were the most correlated parameters (0.5702), while no relationship was observed between M% and melon yield or fruit average weight (−0.1147 and 0.1102, respectively).

**Figure 4:**
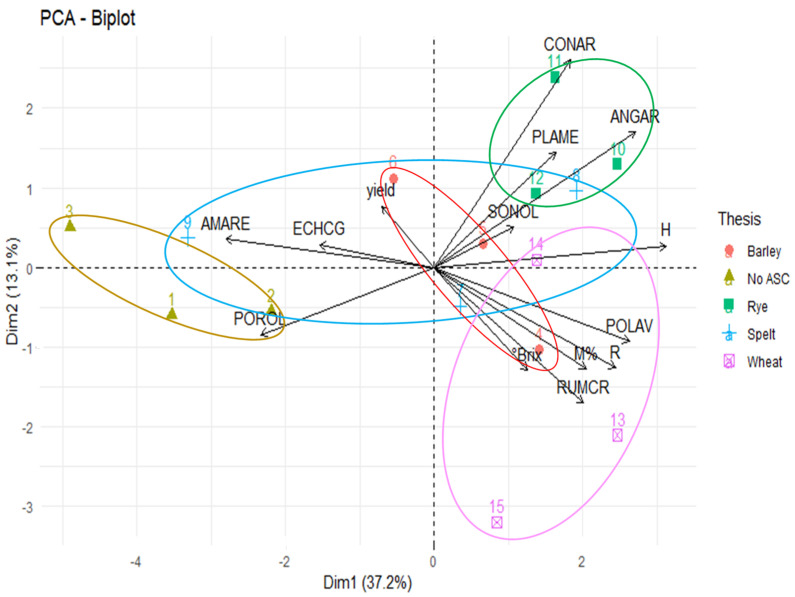
Principal components analysis (PCA) biplot and confidence ellipses ordering the organic melon experimental trial relative to main weed species cover (%), frequency (%) of *Polygonum aviculare* (POLAV), *Anagallis arvensis* (ANGAR), *Rumex crispus* (RUMCR), *Convolvulus arvensis* (CONAR), *Plantago media* (PLAME) and *Sonchus oleraceus* (SONOL), Shannon–Weaver index (H), species Richness (R), melon root mycorrhizal colonization intensity (M%), melon yield and Brix at the August sampling time. Note: No_ASC, fallow.

In relation to the melon sampling at harvest, the PCA extracted two components accounting for 50.3% of the total variability of the whole dataset (the first axis explained 37.2% of the total matrix variance). Ordination of the studied variables along Components 1 and 2 is shown in Figure 4.

The first component completely separated the tilled No ASC control from the no tilled rye and wheat systems, and only partially from the spelt and barley ones. Along the positive values of C1, the no-till ASC systems were associated with the following species: *Polygonum aviculare* L. (POLAV), *Anagallis arvensis* L. (ANGAR), *Rumex crispus* L. (RUMCR) and *Convolvulus arvensis* L. (CONAR), and, secondarily, with *Plantago media* L. (PLAME) and *Sonchus oleraceus* L. (SONOL). These species positively contributed to the global diversity of the sample (H and R diversity indices). Moreover, the ensemble sustains melon mycorrhization and Brix. The species *Amaranthus retroflexus* L. (AMARE) and *Portulaca oleracea* L. (POROL) and, secondarily, *Echinocloa cruss-galli* L. (ECHCG) were correlated with No ASC and uncorrelated with Shannon–Weaver index, species Richness and melon mycorrhization. The melon yield was not correlated with either variables’ groups.

### 3.2. Case-Study on Young Organic Citrus Trees

In Table 1, the list of all weed species found in February and April 2014 sampling in No ASC / Yes ASC systems, their ecological characterization including the SAM trait, are reported. Of a total of 24 weed species found in the field, only three were recognized as no SAM. In Table 3, the frequency of the weed species with mycorrhizal trait (SAM), the weed density, the specie Richness (R), the Shannon–Weaver index, the mycorrhizal colonization intensity of orange tree roots (M), the foliar SPAD and P content, the plant height, the tree canopy diameter and volume, determined in February and April 2014 are reported.

In February, SAM%, weed density, species Richness and Shannon–Weaver index were strongly affected by ASC introduction. The percentage of mycorrhizal weeds was doubled in No ASC system with respect to the Yes ASC ones, along with the weed density, which was significantly lower in Yes ASC system than in No ASC (−54%). Weed richness was significantly higher when barley was introduced with respect to the No ASC system, while the Shannon–Weaver index was higher in the No ASC one compared to the barley one. In April, the % mycorrhizal weeds did not differ between No ASC and Yes ASC systems, while the weed density was five times higher in No ASC (192 plant m^−2^) than in the barley system (41 plant m^−2^). As in February, weed species Richness increased (+80%), while the Shannon–Weaver index correspondingly decreased by about 14% in the Yes ASC system with respect to the No ASC one. The orange mycorrhization increased in the presence of barley, being eight times greater than the No ASC one. Barley intercropping increased the P content of orange tree leaves also (+14%), going from the No ASC-to-ASC system, while SPAD was not influenced by the barley. Other young tree growth parameters were not influenced by the ASC, except the canopy volume which slightly increased (+28%).

**Figure 5:**
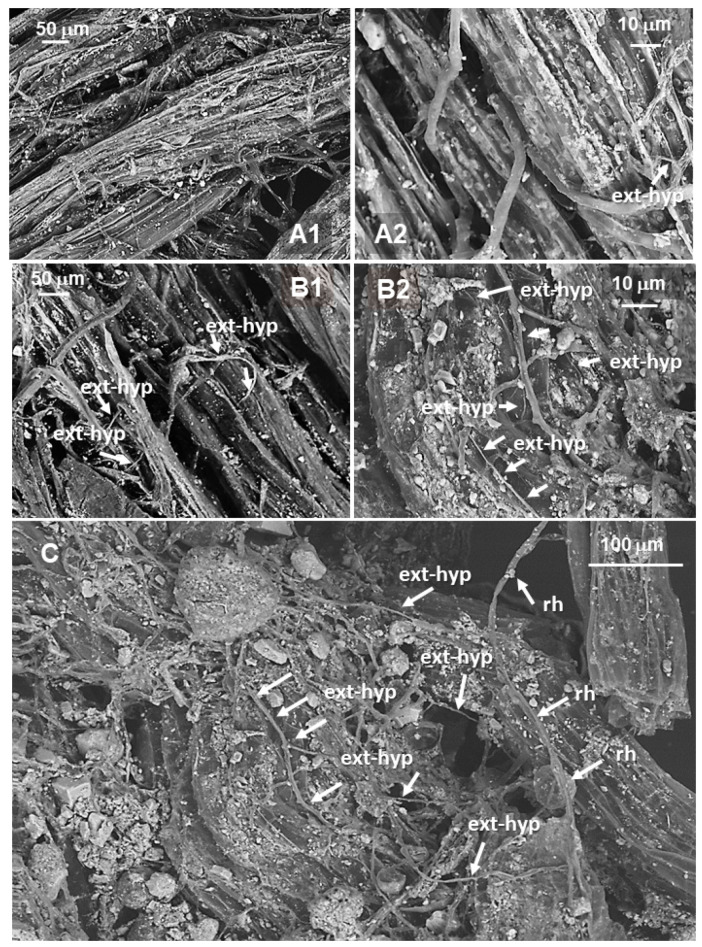
SEM-BSE images of fine lateral roots of orange tree in the No ASC (**A1,A2**) and Yes ASC - barley (**B1,B2**) systems and the mycorrhizal network developed on otrange tree roots inyercropped with barley (**C**). **A1**, **B1**: magnification = 200×; **A2**, **B2**: magnification = 700×; **C**: magnification = 500×. ext-hyp: mycorrhizal extra-radical hyphae; rh: root hairs.

In Figure 5, SEM-BSE and optical microscope images of AMF extra-radical hyphal mycelium (ext-hyp) observed on fine lateral roots of young orange trees sampled in the Yes ASC system intercropped with barley are shown, compared to orange tree roots sampled in the No ASC control system.

Microscopic analyses evidenced more lignified cortex cells on the external surface of orange tree root (Figure 5A1,B1) and mycorrhizal extra-radical hyphae. The abundance of these hyphal external mycelium is coherent with the presence of intercropped barley, able in Yes ASC system to promote the mycorrhizal extra-hyphal development (Figure 5B2) to form a mycorrhizal mycelial network (Figure 5C); it is less evident in orange tree roots in the No ASC system (Figure 5A2).

**Figure 6:**
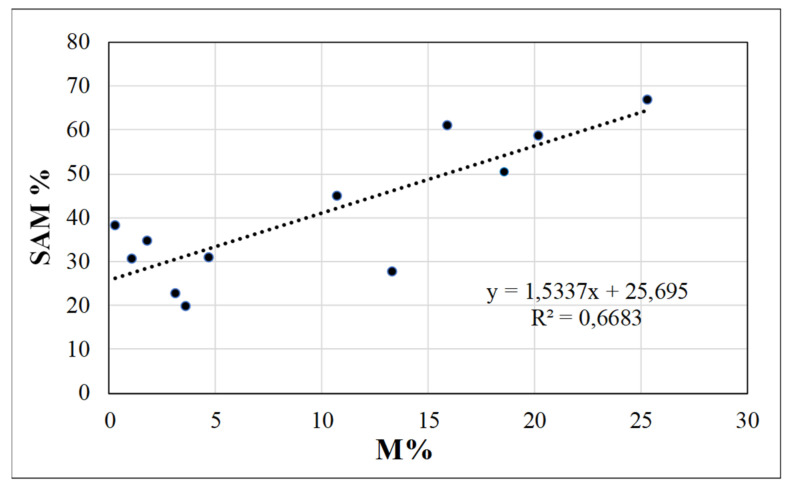
Regression models and *R*^2^ of mycorrhizal colonization intensity (M, in %) vs. frequency of the “Supporting Arbuscular Mycorrhiza” weed species (SAM, in %) in young orange trees systems (No ASC and Yes ASC). Data are refer to April 2014 sampling (N = 12).

In February, correlation analysis of mycorrhizal colonization intensity and other parameters gave no significant correlations among them. On the contrary, in April 2014, orange roots mycorrhization and % mycorrhizal weeds were again significantly correlated (0.587), corresponding to a positive, linear regression (*R*^2^ = 0.6683, Figure 6). The orange roots mycorrhization was negatively correlated to weed richness (−0.558), while Shannon–Weaver index was not significantly correlated (−0.262). No correlation was found among orange roots mycorrhization and specific weed density, while it was positively correlated with foliar P content (0.654) and canopy diameter (0.475).

**Figure 7:**
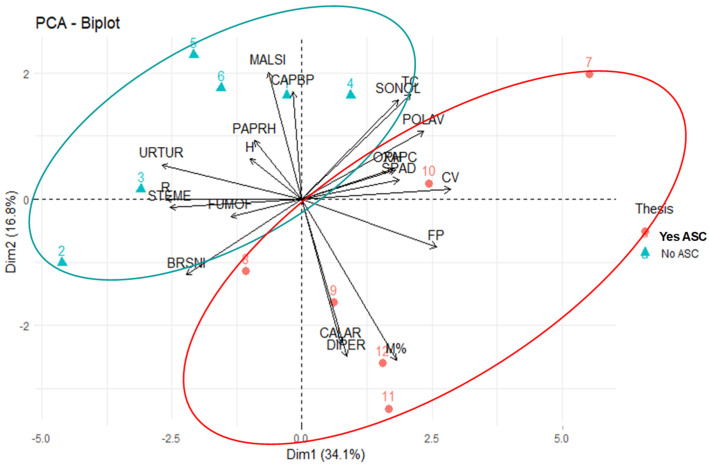
PCA biplot and confidence ellipses ordering the organic orange tree experimental trial relative to main weed species cover (%), frequency (%) of *Polygonum aviculare* (POLAV), *Calendula arvensis* (Vaill.) (CALAR), *Diplotaxis erucoides* (DIPER), *Brassica nigra* (BRNSI), *Stellaria media* (STEME), *Urtica urens* L. (URTUR) and *Malva sylvestris* L. (MALSI), Shannon–Weaver index (H), specie Richness (R), orange root mycorrhizal colonization intensity (M%), foliar SPAD and P content (FP, g kg^−1^), plant height (PH), trunk circumference (TC) and canopy volume (CV) at the April sampling time. No ASC: fallow. Yes ASC: barley.

In relation to the April sampling, the PCA extracted two components accounting for 50.9% of the total variability of the whole dataset (the first axis explained 34.1% of the total matrix variance). Figure 7 reports the ordination of the studied variables along Components 1 and 2.

The Yes ASC system and the No ASC control were completely discriminated along both the two Components. Barley was associated to the species POLAV, the orange tree canopy volume and foliar P along the positive values of C1 and to the species *Calendula arvensis* (Vaill.) L. (CALAR), *Diplotaxis erucoides* (L.) DC. (DIPER) and to the orange tree mycorrhization along the negative values of the C2. Instead, the No_ASC system was associated to the species *Brassica nigra* (L.) W.D.J. Koch (BRSNI), *Stellaria media* L. (STEME), *Urtica urens* L. (URTUR), which sustained the species Richness of the system, along the negative values of the C1, and to *Malva sylvestris* L. (MALSI) along the positive values of C2.

## 4. Discussion

Several studies characterized the plant community based on the relative share of mycorrhizal species in the total vegetation cover or biomass [43,44]. In 2014, Moora [30] proposed to consider the weighted means of plant community mycorrhizal traits by defining the mycorrhization index (MI). More recently, the mycorrhization index of the agroecosystem (MA) was developed, based on a ponderal weight of each plant species within the plant community and its in-field mycorrhizal colonization intensity [14]. The present research proposes an intermediate approach, where the plant mycorrhization measured in field is related to the mycorrhizal functional trait of weeds selected by the different ASC, aiming at verifying the ecological role played by ASC at improving the mycorrhization and yield and/or quality of the cash crop.

The ability of rye and spelt flattened residues in reducing weed density and influence the weed community, due to their allelopathic and competitive potential was duly evidenced [14,19,24,45,46]. In the case-study on organic melon, at harvest (August 2014) the considered ASCs strongly affected the weed community, amplifying the frequency of mycotrophic, mycorrhizal species with an advantage in the weeds–crop competition for soil resources via mycorrhizal symbiosis [36]. At melon harvest, the weed community in Yes ASC was characterized by higher species Richness than the No ASC control, an effect not observed at the June sampling: at the beginning of melon growth, the flattened cereal crops did not only address the weed selection towards mycorrhizal species, but also increased the plant diversity and the melon mycorrhization. This may be due to the ASC mulches, which guaranteed a soil moisture more suitable for fungal community development and, consequently, for mycorrhizal colonization of both the SAM weeds and the cash crop [47].

On the other hand, considering the different ASCs, the AMF extra-radical mycelium observed on melon roots under flattened ASCs, and particularly under wheat, highlighted the ability of mycorrhizal fungi to colonize melon roots, sharing the hyphal network with the neighboring roots of mycorrhizal weeds. Each ASC influenced the mycorrhization of melon and SAM weeds to different extents. In barley system, the mycorrhization of melon and POLAV was the lowest, while in ANGAR it was the same recorded in the rye system and the control: evidently, the flattened barley is not able to boost the mycorrhization of coexisting plants in field. This may be justified by the allelopathic compounds present in flattened barley, such as gramine and hordenine, which can interact with lipid bilayers of plasma membrane of plant root cells, thus inhibiting the AMF colonization of coexisting plants [48,49]. On the contrary, in the wheat system, the mycorrhizal colonization intensity of both the melon and the POLAV, RUMCR and ANGAR was the highest, attesting to the key role played by mycorrhizal weeds under wheat at improving mycorrhization of the cash crop, as further confirmed by PCA. The decrease in mycorrhization observed on SAM weeds in the presence of rye, as in the No ASC system, confirmed the already recognized rye allelopathic properties, which contain the weed growth by inhibiting mycorrhization at their growing first stage [24]. Compared to the control, the spelt increased both the melon mycorrhization and that of RUMCR: evidently, this ASC selected the mycorrhizal weeds in field, such as the SONOL, which was correlated to the melon mycorrhizal infection, as shown by PCA and the positive correlation (0.481) between the cash crop mycorrhization and the frequency of SAM species.

The PCA clearly evidenced the effect of ASC in structuring weed communities: the ASC systems were associated with species cooperating in composing the plant community with a level of diversity higher than the control, as shown by the Shannon–Weaver index and specie Richness correlation pattern. The species habit of the group could help to understand the mechanisms which drove the community dynamics. Indeed, ANGAR, CONAR and POLAV are classified as prostrate/creeping species (Table 1), which are reasonably able to survive the rolling and are well suited to develop between the web of the flattened ASCs [50]. Moreover, according to several studies [46,51] some perennial species, as CONAR, PLAME and RUMCR, may have been encouraged by the no-till conditions. This rich and diverse weed community made up of all mycorrhizal species induced a greater mycorrhization of the melon roots than in the no tilled control by promoting the mycorrhizal network proliferation (Figure 2, Video S1). The tilled control was instead associated to small-weight seed species, such as AMARE and POROL, which negatively correlated with the diversity indices, indicating a dominance condition, also confirmed by the negative correlation between these species and the Shannon–Weaver index. With respect to no-till conditions, the germination and growth of small-seeded annuals in the tilled control may have been favored by greater light availability and less compact soil, and by the absence of the physical growth barrier (i.e., mulch), which prevents radiation and decrease soil surface temperature [46,52,53]. Moreover, AMARE is a non-mycorrhizal species, classified as a highly competitive and nitrophilic weed (C–R species; [54]), which may have easily established a condition of dominance in the weed community of the No ASC system, thus reducing the intensity of the mycorrhizal network in the rhizosphere and, consequently, the melon mycorrhization.

The positive effect of ASCs on weed and cash crop mycorrhization, particularly in wheat system, led to the increase in melon yield and quality in terms of sugar content. In organic horticulture, the positive effect of mycorrhizal bioinoculants application on vegetable crop yield was already observed on different vegetables and fruits such as in watermelon [55], potato [56], or tomato [57]. Conversely, little information is available on the effect of agroecological service crop introduction on the in-field spontaneous mycorrhization in organic production: in our experiment, when ASCs were introduced before melon transplanting, the melon yield increased about 14% with respect to the No ASC system, a value comparable to the 18% observed after application of vesicular arbuscular mycorrhiza (VAM) inoculant in mini-watermelon production [11]. Although the same authors did not record a significant effect of VAM inoculation on fruit weight or total sugar content, in our ASC systems the higher the cash crop mycorrhization was, the higher the melon Brix was, particularly under wheat. This result suggests that mycorrhizal colonization induced by endogenous soil fungal species in the field, favored by ASC mulch, is capable of increasing crop competitiveness in a similar, or even better, way to exogenous inoculation of VAM into soil.

In the case-study on organic orange trees, barley was introduced to contain weeds because it is a fast-growing crop, able to guarantee a high shading and the release of allelochemicals to suppress weed growth [58]. In the case-study on melon, the barley was terminated by flattening and, thus, the allelopathic compounds contained in the mulching layer from barley residues probably drove the mycorrhizal species selection, with the result of reducing the mycorrhization in both the weeds and the melon [59]. Differently, in the orange orchard, barley was intercropped with orange trees, and the study focused on barley before flattening, when the ASC was still alive. In this system, the competition for water and nutrients among barley, the orange tree, and weeds, together with the barley allelopathic attitude, were the elective drivers for SAM weed selection [60]. In fact, while in the melon system the effect of ASCs on mycorrhizal weed frequency, Shannon–Weaver index and specie Richness was observed mainly at crop harvest; in the orange orchard the percentage of weeds able to mycorrhize in Yes ASC system was approximately doubled in February compared to the control, being less relevant in April instead. This supports the hypothesis that the barley interfered with the weeds at the beginning of their growth, selecting them based on their higher competitiveness, mediated by mycorrhization [61]. At April sampling, PCA pointed out the different weed community compositions in the two compared agroecosystems, as shown by the specie Richness correlation pattern; during its vegetative cycle the barley decreased the richness of the weed community, occupying the ecological niches available for spontaneous flora. The N Ellenberg score of the weed species revealed a different degree of nitrophily which characterizes the two No/Yes ASC communities. In particular, except for BRSNI, the species pattern of the control was made up of high N score weed species (STEME, URTUR and MALSI, with N values of eight for all of them), which mean a highly competitive ability in N-rich soils. On the contrary, in the presence of barley, weed species with lower N score values (POLAV, CALAR, DIPER, N value range of 1–5) were predominant: probably the barley, characterized by a depleting effect on nutrients, when introduced as catch crop could have reasonably favored species more suited for soil with low nutrient availability. At the same time, the barley being a mycorrhizal species, it boosted the mycorrhizal network of the system, leading to the greater mycorrhization of the orange roots. It was also found that three of the four weed species (BRSNI, URTUR and MALSI) which characterized the control system were No SAM species, evidencing again the relationship between the mycorrhizal network formed by SAM species and the root mycorrhization of orange trees. When barley was intercropped, the higher orange roots’ mycorrhization determined an improved development of the young orange trees, in terms of foliar P uptake and canopy volume. It should be remarked that many Italian citrus orchards are deficient in P, Zn, Fe and Mn, due to their high clay and lime contents, often coupled with alkaline pH and limited water resources, a condition common to many soils of the Mediterranean region [25]. In these poor soils and under environmental stress, the mycorrhizal symbiosis in citrus roots represent a key to boost the plant nutrient uptake by increasing root absorbing surface [28]. In our citrus orchard, the observed increase in foliar P in presence of barley was positively correlated to the higher orange tree mycorrhization: evidently, the high competition for water, nitrogen and phosphorous with barley pushed the orange tree roots to promote mycorrhizal symbiosis, sustained by the selected mycorrhizal weeds and the mycelial network shared belowground.

In relation to the development of the young tree canopy, once again the higher mycorrhization of citrus roots found in intercropped system corresponds to a higher canopy volume, which must balance the greater development of the mycorrhized root system, in terms of soil volume occupied by tree roots [62].

## 5. Conclusions

We hypothesized that the introduction of ASC can drive weed selection by favoring the mycorrhizal weeds, thereby increasing the cash crop root mycorrhization and positively influencing its nutrient uptake, growth, yield and/or quality.

The observed ASC–weed–AMF interactions showed the possibility of selecting the most performing agroecological service crop able to indirectly promote the cash crop mycorrhization via sharing the mycorrhizal mycelial network: this offers a great opportunity to intensify the plant–AMF interactions belowground and enhance the conservation of agroecosystems long-term. The no-tillage, green mulches and intercropping appear as effective methods to manage the organic cropping systems well [63] to promote beneficial fungi growth and emphasize the plant–fungi symbiosis.

In accordance with our work hypothesis, the positive effect of the ASCs is not only related to the promotion of beneficial plant–microorganism interactions but also to the increase in yield and quality of the cash crop: this is a key aspect for farmers when introducing sustainable practices within the cropping systems and a relevant economic factor to be considered when assessing the overall functionality of the agroecosystem.

## Figures and Tables

**Table 1:** Ecological characterization of weed species found in the vegetable (melon) and orange systems at the two sampling times per system. The cross symbol identifies those with the positive Supporting Arbuscular Mycorrhizal (SAM) trait [36].

Species	EPPO ^1^ code	Raunkiaer ^2^ BG	Habit	N ^12^	SAM	Vegetable System		Orange System		Reference
						June	Aug.	Feb.	Apr.	
*Aster squamatus*	ASTSQ	T ^3^ Scap ^7^	Erect	7		*	*			
*Amaranthus retroflexus*	AMARE	T Scap	Erect	9		*	*	*	*	
*Anagallis arvensis*	ANGAR	T Rept ^8^	Prostrate	6	X	*	*	*	*	A
*Beta vulgaris*	BETVU	H ^4^ Scap	Rosette	5				*	*	
*Bromus sterilis*	BROST	T Scap	Erect	5	X			*	*	A
*Brassica nigra*	BRSNI	T Scap	Erect	4				*	*	
*Calendula arvensis*	CALAR	T Scap	Erect	5	X			*	*	A
*Capsella bursa-pastoris*	CAPBP	H Bienne ^8^	Rosette	4				*	*	
*Chondrilla juncea*	CHOJU	H ^4^ Scap	Erect	x	X	*	*			A
*Convolvolus arvensis*	CONAR	G ^5^ Rhiz ^9^	Prostrate/creeping	5	X	*	*	*	*	A
*Conyza canadensis*	ERICA	T Scap	Erect	7	X	*	*			A
*Diplotaxis erucoides*	DIPER	T Scap	Erect	5				*	*	
*Echinochloa crus-galli*	ECHCG	T Scap	Erect	8		*	*			
*Fumaria officinalis*	FUMOF	T Scap	Erect	6				*	*	
*Glebionis segetum*	CHYSE	T Scap	Erect	5	X			*	*	A
*Helminthotheca echioides*	PICEC	T Scap	Erect	2	X	*	*			A
*Lamium amplexicaule*	LAMAM	T Scap	Erect	7				*	*	
*Lolium perenne*	LOLPE	H Caesp ^10^	Erect	7	X	*	*			A
*Malva silvestris*	MALSI	H Scap	Erect	8				*	*	
*Mercurialis annua*	MERAN	T Scap	Erect	8	X			*	*	A
*Oxalis pes-caprae*	OXAPC	G Bulb ^10^	Prostrate	5				*	*	
*Papaver rhoeas*	PAPRH	T Scap	Erect	x	X			*	*	A
*Plantago media*	PLAME	H Ros ^11^	Rosulate	3	X	*	*			A
*Poa annua*	POAAN	T Caesp	Erect	8	X	*	*			A
*Polygonum aviculare*	POLAV	T Rept	Prostrate/creeping	1	X	*	*	*	*	B
*Portulaca oleracea*	POROL	T Scap	Prostrate/creeping	7	X	*	*			A
*Raphanus raphanistrum*	RAPRA	T Scap	Erect	5				*	*	
*Rumex crispus*	RUMCR	H Scap	Erect	5	X	*	*			B
*Senecio vulgare*	SENVU	T Scap	Erect	8	X			*	*	A
*Setaria viridis*	SETVI	T Scap	Erect	7	X	*	*			A
*Sonchus oleraceus*	SONOL	T Scap	Erect	6	X	*	*	*	*	A
*Stellaria media*	STEME	T Rept	Prostrate	8	X	*	*	*	*	B
*Trifolium repens*	TRFRP	Ch ^6^ Rept	Prostrate	7	X	*	*			C
*Urtica urens*	URTUR	T Scap	Erect	8				*	*	
*Verbascum sinuatum*	VESSI	H Bienne	Rosette	7				*	*	
*Veronica persica*	VERPE	T Scap	Prostrate	6	X	*	*	*	*	A

^1^ European and Mediterranean Plant Protection Organization; ^2^ Raunkiaer biological group; ^3^ Therophytes; ^4^ Hemicryptophytes; ^5^ Geophytes; ^6^ Chamaephyta; ^7^ Scapose; ^8^ Reptant; ^9^ Rhizomatous; ^10^ Caespitose; ^11^ Rosulate; ^12^ Ellenberg N-score. Asterisks indicate the presence of the species in the associated experimental year. A: [36]: B: [14]; C: [42].

**Table 2:** Frequencies of species with confirmed mycorrhizal association (SAM%), weed density (pp m^−2^), weed cover (%, on m^−2^), species Richness (R), Shannon–Weaver index (H), mycorrhizal colonization intensity of melon (M%), melon yield (Mg ha^−1^), average fruit weight (kg fruit^−1^), melon sugar content (Brix), recorded in 2014 at melon ripening and/or at melon harvest. ASC: agroecological service crop. Levels of statistical significance (p-value) are: * *p* < 0.05, ** *p* < 0.01 and *** *p* < 0.001 (ANOVA). Different letters represent significant differences (Tukey’s HSD test for means comparison).

Melon												
	June 2014 (Melon Ripening)				August 2014 (Melon Harvesting)							
	SAM (%)	Density (pp m^−2^)	R	H	SAM (%)	Cover (%)	R	H	M%	Yield (Mg ha^−1^)	Fruit Weight (kg fruit^−1^)	Sugar Content (Brix)
**ASC**												
No	66.3	345 a	4.3	0.90 b	40.8 b	87.5	6.3 b	1.15 b	15.9 b	10.5 b	1.5	7.4 b
Yes	44.4	196 b	5.7	1.36 a	83.6 a	79.1	9.5 a	1.65 a	24.5 a	12.2 a	1.2	8.0 a
*Sig.*	*n.s.*	**	*n.s.*	*	***	*n.s.*	*	*	**	*	*n.s.*	*
**ASC spec.**												
Barley	42.7	238 ab	6.3	1.33	73.6	79.2	10.0 ab	1,63	17.6 b	8.27	0.97	8.07
Rye	49.6	139 b	5.3	1.63	91.8	79.2	8.3 b	1,77	27.0 ab	13.76	1.17	7.95
Spelt	46.8	116 b	4.7	1.23	84.6	70.8	8.0 b	1,44	20.7 b	16.22	1.49	7.75
Wheat	38.6	292 a	6.3	1.37	84.4	87.5	11.7 a	1,76	32.8 a	10.69	1.32	8.45
*Sig.*	*n.s.*	**	*n.s.*	*n.s.*	*n.s.*	*n.s.*	*	*n.s.*	*	*n.s.*	*n.s.*	*n.s.*

**Table 3:** Frequencies of species with confirmed mycorrhizal association (SAM%), weed density (pp m^−2^), specie Richness (R), Shannon–Weaver index (H) and mycorrhizal colonization intensity (M%), foliar SPAD, foliar P (g kg^−1^), plant height (cm), canopy diameter (cm) and canopy volume (m^3^), recorded in 2014 on young orange trees organic production system. ASC: agroecological service crop (barley). Levels of statistical significance (*p*-value) are: * *p* < 0.05, ** *p* < 0.01 and *** *p* < 0.001 (ANOVA). Different letters represent significant differences (Tukey’s HSD test for means comparison).

Young Orange Trees														
	February 2014 (DAS 135)				April 2014 (DAS 187)									
	SAM(%)	Density (pp m^−2^)	R	e	SAM (%)	Density (pp m^−2^)	R	H	M%	SPAD	Foliar P (g kg^−1^)	Plant Height (cm)	Canopy Diameter (cm)	Canopy Volume (m^3^)
**ASC**														
No	40.8 a	396 a	5.3 b	0.77 a	35.4	192 a	3.6 b	1.8	2.4 b	74.37	1.50 b	157	88	0.65 b
Yes	18.7 b	238 b	7.7 a	0.64 b	43.7	41 b	6.5 a	1.5	17.2 a	75.59	1.71 a	164	95	0.83 a
*Sig.*	***	***	**	***	*n.s.*	***	*	*n.s.*	***	*n.s.*	**	*n.s.*	*n.s.*	*

## Data Availability

The data presented in this study are available on request from the corresponding author.

## Supplementary Materials

The following are available online at https://www.mdpi.com/2076-2607/9/2/410/s1: Video S1: Mycorrhizal mycelial network observed on melon root fragment sampled in the ASC (wheat) system after staining by 0.05% *w/v* methyl blue in lacto-glycerol solution and Scanning Electron Microscopy under Variable Pressure (SEM-VP) analysis of the root fragments (chamber pressure: 10 Pa; WD: 10 mm). (a): Mag. 250X; (b): mag. 500X; (a): mag. 1.0 KX; (a): mag. 2.0 KX (re: root epidermis; mn: mycorrhizal mycelial network; ext-hyp; external hyphae; rh: root hair).
